# Comparison of different oval window sealing materials in stapes surgery: systematic review and meta-analysis

**DOI:** 10.1007/s00405-022-07551-z

**Published:** 2022-07-20

**Authors:** Alfonso Scarpa, Pasquale Marra, Massimo Ralli, Pasquale Viola, Federico Maria Gioacchini, Giuseppe Chiarella, Francesco Antonio Salzano, Pietro De Luca, Filippo Ricciardiello, Claudia Cassandro, Grazia Maria Corbi

**Affiliations:** 1 Department of Medicine, Surgery and Dentistry, University of Salerno, Largo Città di Ippocrate, 84131 Salerno, Italy; 2 Department of Sense Organs, Sapienza University of Rome, Rome, Italy; 3 Department of Experimental and Clinical Medicine, Unit of Audiology, Regional Centre for Cochlear Implants and ENT Diseases, Magna Graecia University, Catanzaro, Italy; 4 ENT Unit, Department of Clinical and Molecular Sciences, Polytechnic University of Marche, Ancona, Italy; 5 Ear, Nose, and Throat Unit, AORN “Antonio Cardarelli”, 80131 Naples, Italy; 6 Department of Surgical Sciences, University of Turin, Turin, Italy; 7 Department Medicine and Health Sciences, University of Molise, Campobasso, Italy

**Keywords:** Stapes, Stapes surgery, Sealing, Oval window, Sealant

## Abstract

**Objective:**

To compare the efficacy and safety characteristics of different materials used for oval window sealing during stapedotomy.

**Methods:**

A systematic review was conducted according to the PRISMA guidelines. Published international English literature from January 1, 2000 to December 2021 was screened, checking for studies that compared different materials utilization in patients undergoing stapedotomy surgery for otosclerosis or congenital stapes fixation. Data related to the efficacy and safety of each material were extracted. The primary outcome measure was the air–bone gap (ABG) closure after surgical intervention.

**Results:**

Six studies were included in the metanalysis. Because of the heterogeneity of the treatments adopted, we assessed the use of the fat compared to all other treatments, and the use of the gelfoam compared to all other treatments. In the former analysis (fat vs others) we did not identify differences in ABG closure between the groups (*p* = 0.74), with a low heterogeneity of the results (*I*^2^ = 28.36%; Hedge’s *g* = 0.04, 95% CI − 0.19 0.27); similarly, we did not identify differences between the use of gelfoam and other treatments (*p* = 0.97), with a low heterogeneity of the results (*I*^2^ = 28.91%; Hedge’s *g* = 0.00, 95% CI − 0.20 0.21).

**Conclusions:**

Numerous options are available for oval window sealing during stapedotomy, with acceptable safety and effectiveness profiles. Based on the current data, no definitive recommendation can be made regarding the choice of one material over another, and the convenience of sealing over no sealing at all.

## Introduction

Stapes surgery has changed considerably over time. Shea was the first to perform a stapedectomy in 1956 covering the oval window with a thin slice of subcutaneous tissue, and reconstructing the sound-conducting mechanism of the middle ear with a Teflon replica of the stapes [1]. Later, other techniques such as partial stapedectomy and stapedotomy were developed. Sealing the oval window during stapes surgery is a widely used practice, especially when stapedectomy was performed to preserve the inner ear and reconstruct the sound-conducting mechanism [2]. On the contrary, its role in stapedotomy is less clear, and, according to some authors, it would be useful only when the fenestration is made too large [3]. Sealing the oval window after fenestra would improve the sound conduction [4], preclude the formation of a labyrinthine fistula, and act as a barrier to infections [5].

Different sealing materials are reported in the literature, both autologous and heterologous, with the former being the most used [6]. Among the materials, adipose tissue, perichondrium, vein graft, temporalis fascia, blood clot, hyaluronic acid, and gelatin sponge are the most used, with acellular porcine-derived matrix [5, 7].

However, there is a lack of consensus about sealing or not-oval window after fenestration and possibly which material to use [8, 9]. Some materials could be harmful causing fibrous reactions, granuloma formation, and toxic effects on the inner ear [10].

The objective of this systematic review was to revise the literature on the effect of different sealing materials in primary stapedotomy for otosclerosis measured by hearing outcome, sensorineural hearing loss (SNHL), and postoperative dizziness.

Moreover, with a metanalysis, we tried to evaluate the effects of different sealing materials on the postoperative closure of the air–bone gap (ABG).

We tried to answer the following questions: does oval window sealing positively affect postoperative auditory performance? Does one material have a particular advantage over the others?

## Materials and methods

### Study design

This study was conducted according to the Preferred Reporting Items for Systematic Reviews and Meta-Analyses Statement [11], and the study protocol was registered on PROSPERO (reference number CRD42022304958) without subsequent modifications to the drafting available at https://www.crd.york.ac.uk/prospero/display_record.php?RecordID=304958

### Search strategy

We carried out a literature search on Pubmed/MEDLINE, Cochrane, EMBASE, and OVID databases for articles published from December 2001 to 31st December 2021, in English. We also performed a snowball search to identify additional studies by searching the reference lists of publications eligible uploaded to EndNote (Clarivate Analytics, Philadelphia, PA). The search string was the following: “Stapes Surgery” [Mesh] “Otosclerosis”[Mesh] “Progressive hearing loss stapes fixation” [Supplementary Concept] “stapedectomy” [tw] “stapedotomy” [tw] “Oval Window, Ear”[Mesh], “Tissue Transplantation”[Mesh] “Connective Tissue” [Mesh] “Gelatin Sponge, Absorbable”[Mesh], “Fascia”[Mesh], “Veins”[Mesh] “Hyaluronic Acid”[Mesh] “Seal”[tw] “Sealing”[tw] “Graft”[tw] “Vein”[tw] “Fat”[tw]. Two authors (A.S., P.M.) independently reviewed the titles and abstracts obtained from all the databases. Then, the full texts were assessed. All studies that did not meet the inclusion criteria and duplicates were excluded. Cross-references were verified, and valuable articles were included. In the case of disagreement about the eligibility of a study, a third author (M.R.) decided which articles were included. Data were extracted independently by two authors (A.S., P.M.) and confirmed where necessary by the principal investigator (A.S.). A data extraction spreadsheet was developed and the information from the studies included in the review was extracted and tabulated using an Excel sheet, double-checked for accuracy. Another reviewer (M.R.) checked the data collected.

### Study selection criteria

The study selection was based on the Population, Intervention, Comparison, Outcome, and Study (PICOS) framework [12] (Table 1). The air–bone gap (ABG) closure (post-operative air conduction minus postoperative bone conduction) was defined according to the American Academy of Otolaryngology-Head and Neck Surgery (AAO-HNS) [13]. The calculation was made by using the four-tone average (0.5–1.3, 3, or 4 kHz). Surgical success is usually defined as the post-operative closure of the ABG to ≤ 10 dB, without deterioration of bone conduction (BC) at all frequencies [14].

**Table 1:** PICOS framework

**P**opulation	Patients undergoing stapedotomy surgery for otosclerosis or congenital stapes fixation
**I**ntervention	Use of fat or gelfoam as sealing materials
**C**omparison	No use of fat or gelfoam as sealing materials
**O**utcome	Air–Bone Gap closure
**S**tudy	Comparative randomized or non-randomized trials in English

Studies that considered stapedectomy, ossiculoplasty, or cases of surgical revision were excluded. We excluded reviews, case reports, case series, letters, comments, and editorials. Articles that did not satisfy the primary outcome were excluded. Secondary outcomes included SNHL and postoperative dizziness. All the outcomes were assessed at the longest follow-up time.

### Data extraction

Two review authors (AS and PM) independently extracted data and assessed the risk of bias for selected studies including the population demographics, study design, type of surgical procedure, type of prosthesis used, outcome, and follow-up. Discrepancies were identified and resolved through discussion.

### Statistical analysis

The meta-analysis was carried out using the STATA 16 v statistical software, and a *p* value ≤ 0.05 was considered to be statistically significant. Based on the treatment, we performed the first analysis stratifying the population by the use of fat compared to all other treatments. The second analysis was performed considering, as treatment, the use of the gelfoam. Data were expressed as mean ± standard deviation with a 95% confidence interval. The summary statistics required for each outcome were the number of participants in the treatment and control groups at post-test, and the mean and SD of the ABG closure outcome after the intervention. Random-effects models were prespecified a priori, given the heterogeneity across settings, participants, and sample size [15].

Statistical heterogeneity was calculated using the Higgins *I*^2^ statistic, which describes the percentage of variability in the effect estimate due to heterogeneity rather than sampling error. Inconsistency was examined using *I*^2^ and the following grades were applied: < 25% (very low), 25 to < 50% (low), 50 to < 75% (moderate), and ≥ 75% (large) [16, 17]. The possibility of small study effects was assessed qualitatively by a visual estimate of the Funnel plot and quantitatively by calculation of the Egger and Begg’s tests [18, 19].

Besides, we performed subgroup analysis to test interactions according to the study design (retrospective, RCT). A Meta-regression was also performed to check the possible influence of confounders (e.g., age and gender) on the results.

## Results

### Selection and studies inclusion

**Fig. 1:**
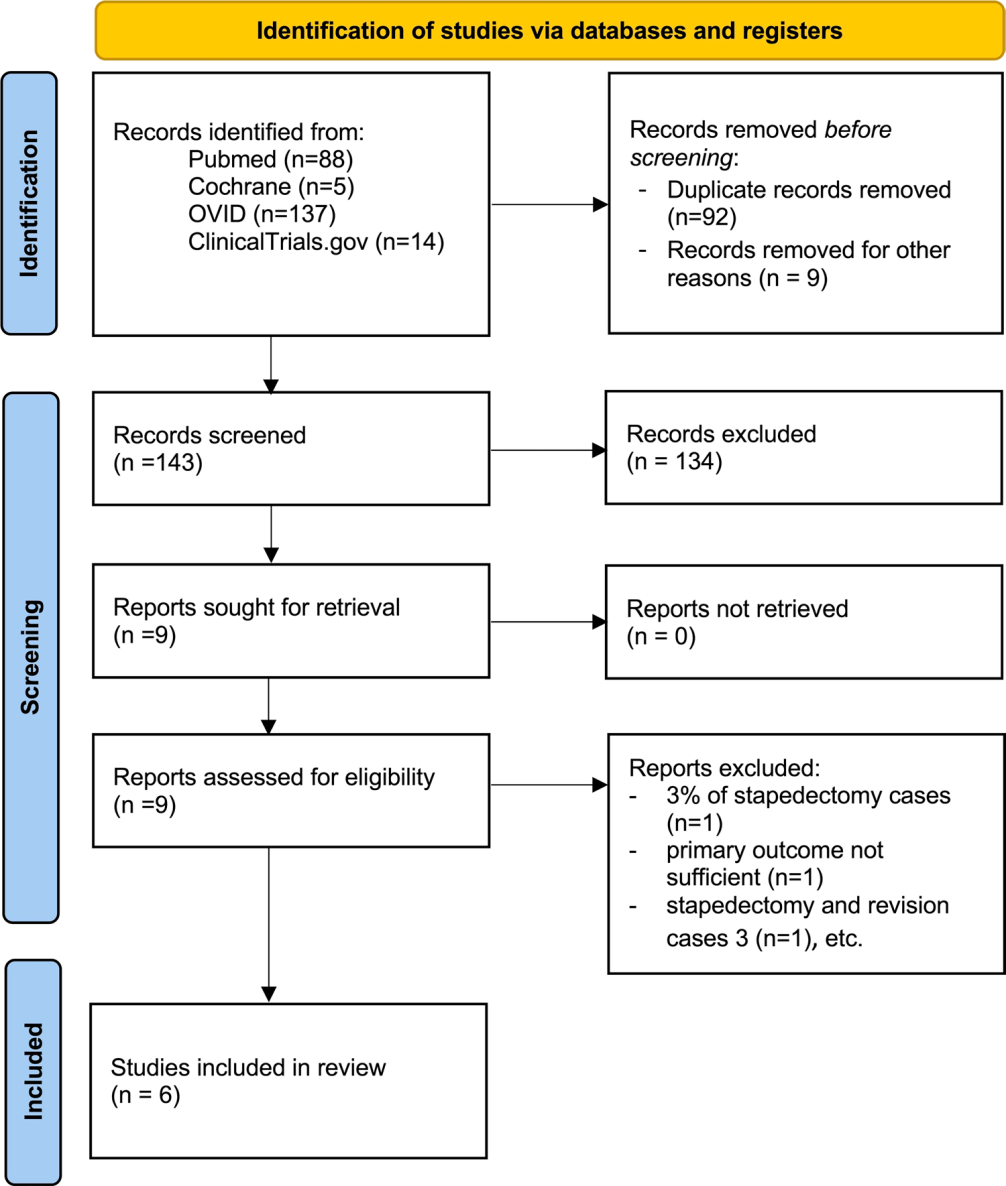
PRISMA algorithm

The database search yielded 244 results. Figure 1 shows the PRISMA algorithm. Six studies were included in the review [20–25]. Characteristics of the studies are summarized in Table 2.

**Table 2:** Characteristics of the studies included in the metanalysis

Study	Population and treatment	Follow-up (months)	ABG closure	*p* value
Angeli (2006)	27 pts received 0.2 ml of HA before fenestration and subsequently adipose tissue grafts around the piston 32 patients received only adipose tissue grafts around the piston	15.5–13.7	6.6 ± 5.2 dB for the HA 7.6 ± 5.3 for the no HA group (*p* = 0.4)	0.4
Faramarzi (2019)	86 patients received an adipose graft 90 patients received a gelfoam graft	6	10.2 ± 6.7 for the adipose group 11.3 ± 8.6 for the gelfoam group	0.532
Bawazeer (2020)	215 patients received the gelfoam to seal the oval window after fenestration 203 control patients (without sealing)	12	4.6 ± 5.7 in the gelfoam group 5.3 ± 6.9 in the control group	0.634
Faramarzi (2021)	73 patients received 0.5 ml HA around the piston and in the middle ear cavity, 73 patients received lobule fat graft around the piston	6	14.6 ± 7.2 dB for the HA 16.3 ± 6.7 dB for the fat	0.337
Pradhan (2020)	26 patients received adipose tissue in sealing the oval window after insertion of the piston, 25 patients received gelfoam in sealing the oval window after insertion of the piston	3	13.92 ± 6.8 dB in the adipose group 15.42 ± 7.5 dB in the gelfoam group	0.56
Schmerber (2004)	314 subjects received vein graft interposition 128 subjects received tragal perichondrium	4	4.88 ± 5.77 dB in the adipose group 6.23 ± 6.10 dB in the gelfoam group	0.614

Two studies were from Iran and one each from the United States, France, Saudi Arabia, and India. Of the six studies, two were randomized control trials and four were retrospective cohort studies. A total of 1298 patients were evaluated, with a mean age of 39.9 ± 6.27 years, and a female to male ratio of 1.5:1.

### Study risk of bias assessment

The methodological quality of included studies was checked independently by two authors (AS and PM) using the Downs and Black checklist [26]. It has been ranked in the top six quality assessment scales suitable for use in systematic reviews [27]. The checklist consists of 27 items to assess the quality of reporting, external validity, internal validity (bias and confounding) and power. As in other systematic reviews [28, 29], the tool was modified slightly for use in this review simplifying the scoring for question 27 dealing (statistical power) to assign either 1 point or 0 rather than a score from 0 to 5. A score of 1 was assigned to studies that included a power calculation, while a score of 0 was given to studies without any power calculation.

Downs and Black score ranges were grouped into the following 4 quality levels: excellent [26–28], good [20–25], fair [15–19], and poor [14].

Any disagreements were discussed until a consensus was reached or resolved by a third author (MR). Two studies were evaluated good, two were evaluated fair and one was evaluated poor (Table 3)*.*

**Table 3:** Quality assessment of the included studies using the Downs and Black checklist

	Angeli 2006	Bawazeer 2020	Faramarzi 2019	Faramarzi 2020	Pradhan 2020	Schmerber 2004
Q1: Aim clearly described?	Yes	Yes	Yes	Yes	Yes	Yes
Q2: Outcomes clearly described?	Yes	Yes	Yes	Yes	No	Yes
Q3: Patients’ characteristics clearly described?	Yes	Yes	Yes	Yes	Yes	No
Q4: Interventions clearly described?	Yes	Yes	Yes	Yes	Yes	Yes
Q5: Principal confounders clearly described?	No	No	No	No	No	No
Q6: Main findings clearly described?	Yes	Yes	Yes	Yes	Yes	Yes
Q7: Random variability for main outcome provided?	Yes	Yes	Yes	Yes	Yes	No
Q8: Adverse events reported?	Yes	No	No	No	Yes	No
Q9: Loss-to-follow up reported?	Yes	Yes	Yes	Yes	Yes	Yes
Q10: Actual p-value reported?	Yes	Yes	Yes	Yes	Yes	Yes
Q11: Sample asked to participate representative of the population?	No	Yes	Yes	Yes	No	No
Q12: Sample agreed to participate representative of the population?	No	No	No	No	No	No
Q13: Staff participating representative of the patients’ environment?	No	Yes	Yes	Yes	Yes	Yes
Q14: Attempt to blind participants?	No	No	Yes	Yes	No	No
Q15: Attempt to blind assessors?	No	No	Yes	Yes	No	No
Q16: Data dredging results stated clearly?	Yes	Yes	Yes	Yes	Yes	Yes
Q17: Analysis adjusted for length of follow up?	No	No	Yes	Yes	No	Yes
Q18: Appropriate statistics?	Yes	Yes	Yes	Yes	Yes	No
Q19: Reliable compliance?	Yes	Yes	Yes	Yes	Yes	Yes
Q20: Accurate outcome measures?	Yes	Yes	Yes	Yes	Yes	No
Q21: Same population?	Yes	Yes	Yes	Yes	Yes	No
Q22: Participants recruited at the same time?	No	No	Yes	Yes	Yes	No
Q23: Randomised?	No	No	Yes	Yes	No	No
Q24: Adequate allocation concealment?	No	No	Yes	Yes	No	No
Q25: Adequate adjustment for confounders?	No	Yes	No	No	No	No
Q26: Loss of follow up reported?	Yes	Yes	Yes	Yes	Yes	Yes
Q27: Power calculation?	No	No	Yes	Yes	No	No

### Study characteristics

In the included studies, there were 327 patients in the gelfoam group, 314 in the tragal perichondrium group, 203 in the no graft group, 184 in the lobule fat group, 138 in the vein graft group, 100 in the hyaluronic acid group, and 32 in the adipose graft tissue around the piston group. A difference in the ABG calculation method was observed: Angeli’s [20], Pradhan's [24], and Schmerber’s [25] studies used the means of the thresholds for bone and air conduction at 0.5, 1, 2, and 4 kHz, while Faramarzi [22], Bawazeer [21], and Faramarzi [23] adopted 0.5, 1, 2, and 3 kHz. The latter was calculated as an average of 2 and 4 kHz frequencies. Because of the heterogeneity of the studies included, we decided to focus our investigation on fat versus other materials and gelfoam versus other materials.

### Primary outcome

In our study, we identified as primary outcome the ABG after the intervention. Because of the heterogeneity of the treatments adopted, we checked two main methods: the use of fat compared to all other treatments, and the use of the gelfoam compared to all other treatments.

**Fig. 2:**
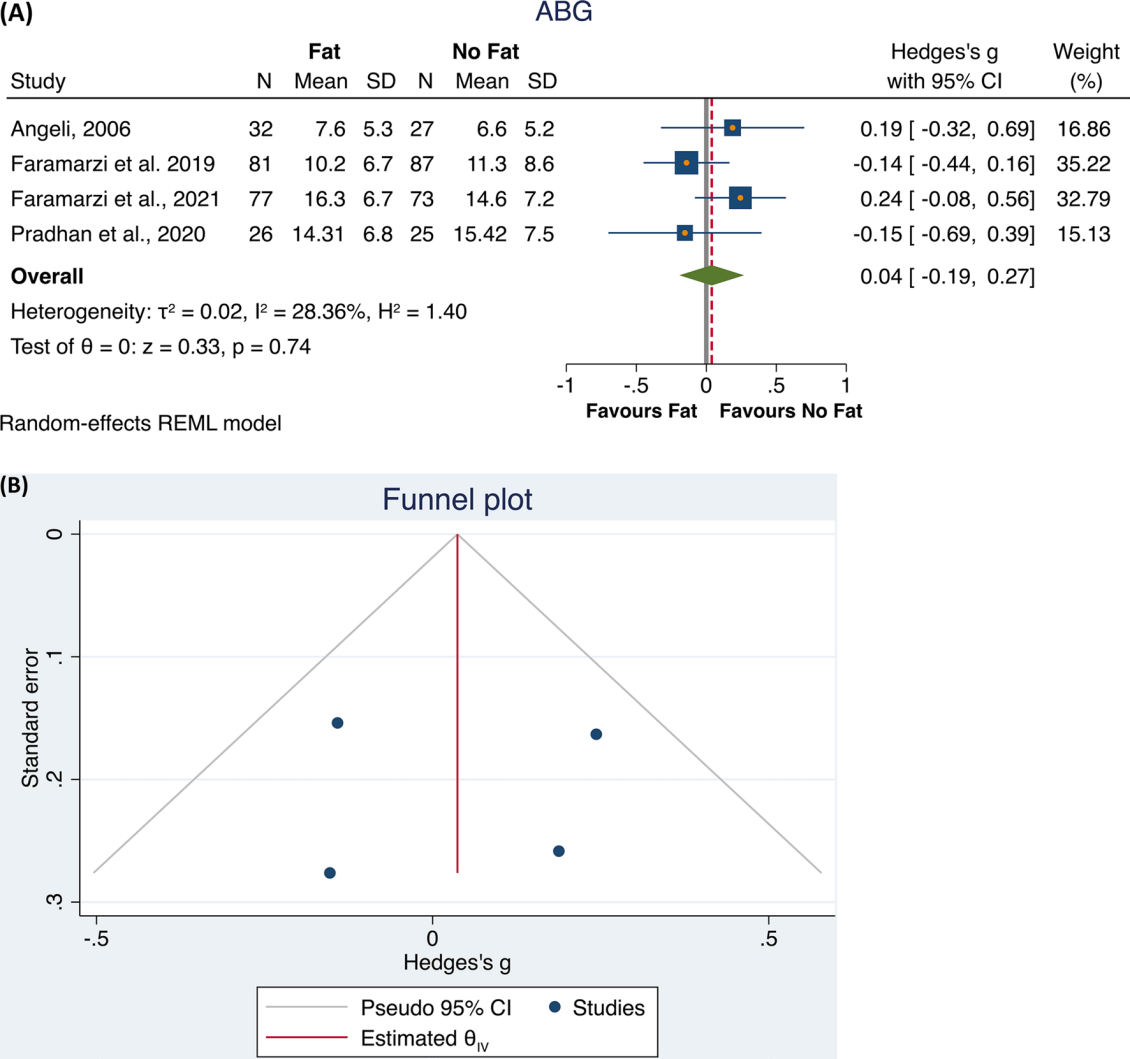
Forest plot (**A**) and Funnel plot (**B**) of the studies comparing the fat and all other treatments in ABG after the intervention

**Fig. 3:**
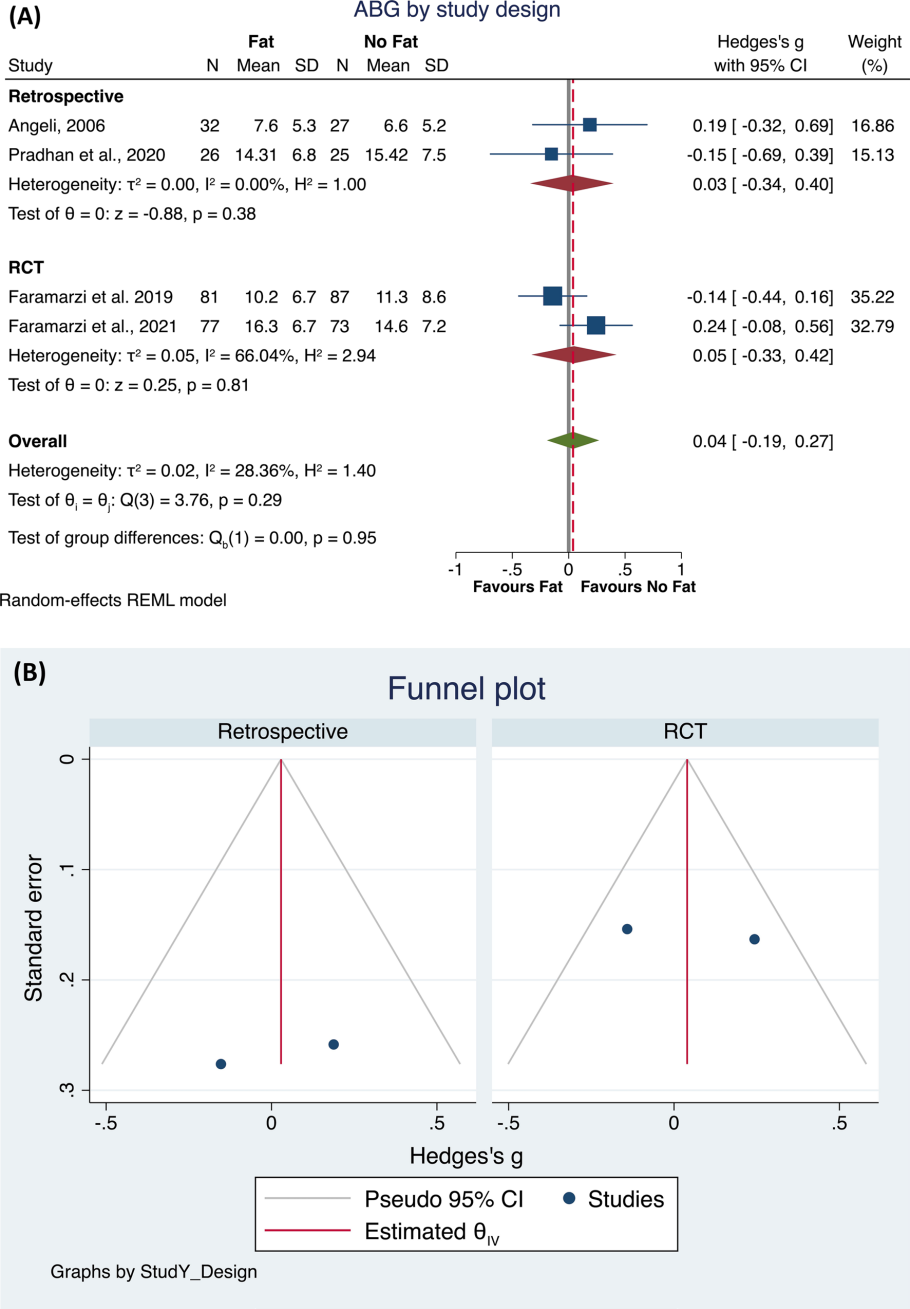
Forest plot (**A**) and Funnel plot (**B**) of the subgroup analysis by Retrospective or RTCs study design in studies comparing the fat and all other treatments in ABG after the intervention,

The first analysis found that four studies [20, 22–24] were eligible. The total population included 216 subjects who underwent a surgical procedure using fat, compared to 212 subjects who underwent a surgical procedure not using fat. The meta-analysis did not identify differences between the groups (*p* = 0.74), with a low heterogeneity of the results (*I*^2^ = 28.36%; Hedge’s *g* = 0.04, 95% CI − 0.19 0.27; Fig. 2A). The Funnel plot and the Egger and Begg’s test did not show small study effects publication bias (Fig. 2B). Then, to check the possible interaction of the study design with these results, we performed a sub-analysis. Both in retrospective and RTC studies, no statistically significant differences were found in ABG closure between the two groups, whereas in the retrospective studies a very low heterogeneity was found (*I*^2^ = 0.0%, *p* = 0.38), while the RTCs had a moderate heterogeneity (*I*^2^ = 66.04%, *p* = 0.81). Noteworthily both the subgroups included only two studies (Fig. 3A, B).

Then, investigating the possible effect on post-operative ABG of the use or not of gelfoam, we found only three studies eligible [21, 22, 24]. The total population included 327 subjects who underwent a surgical procedure using gelfoam, compared to 310 subjects who underwent a surgical procedure not using gelfoam.

**Fig. 4:**
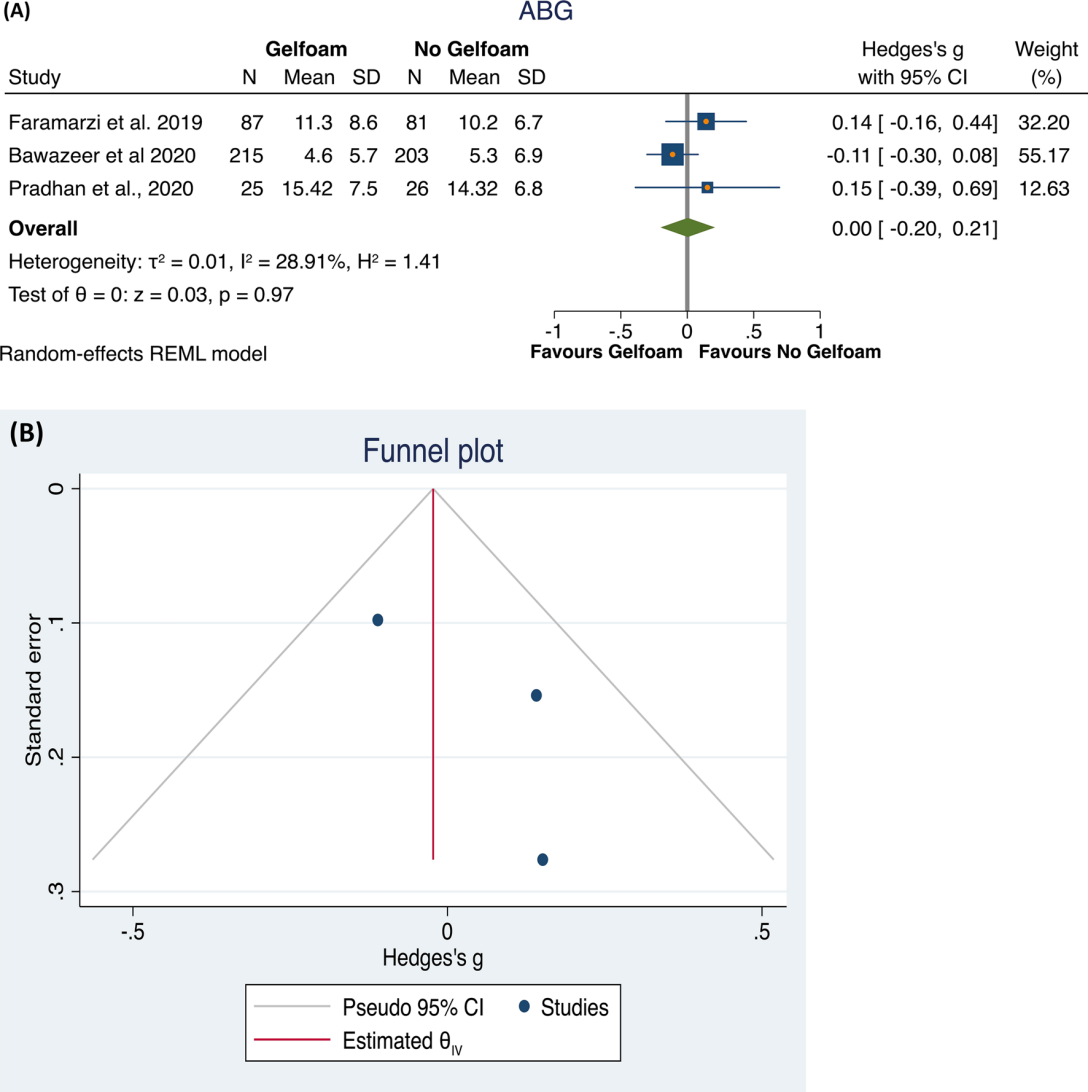
Forest plot (**A**) and Funnel plot (**B**) of the studies comparing the gelfoam and all other treatments in ABG after the intervention

**Fig. 5:**
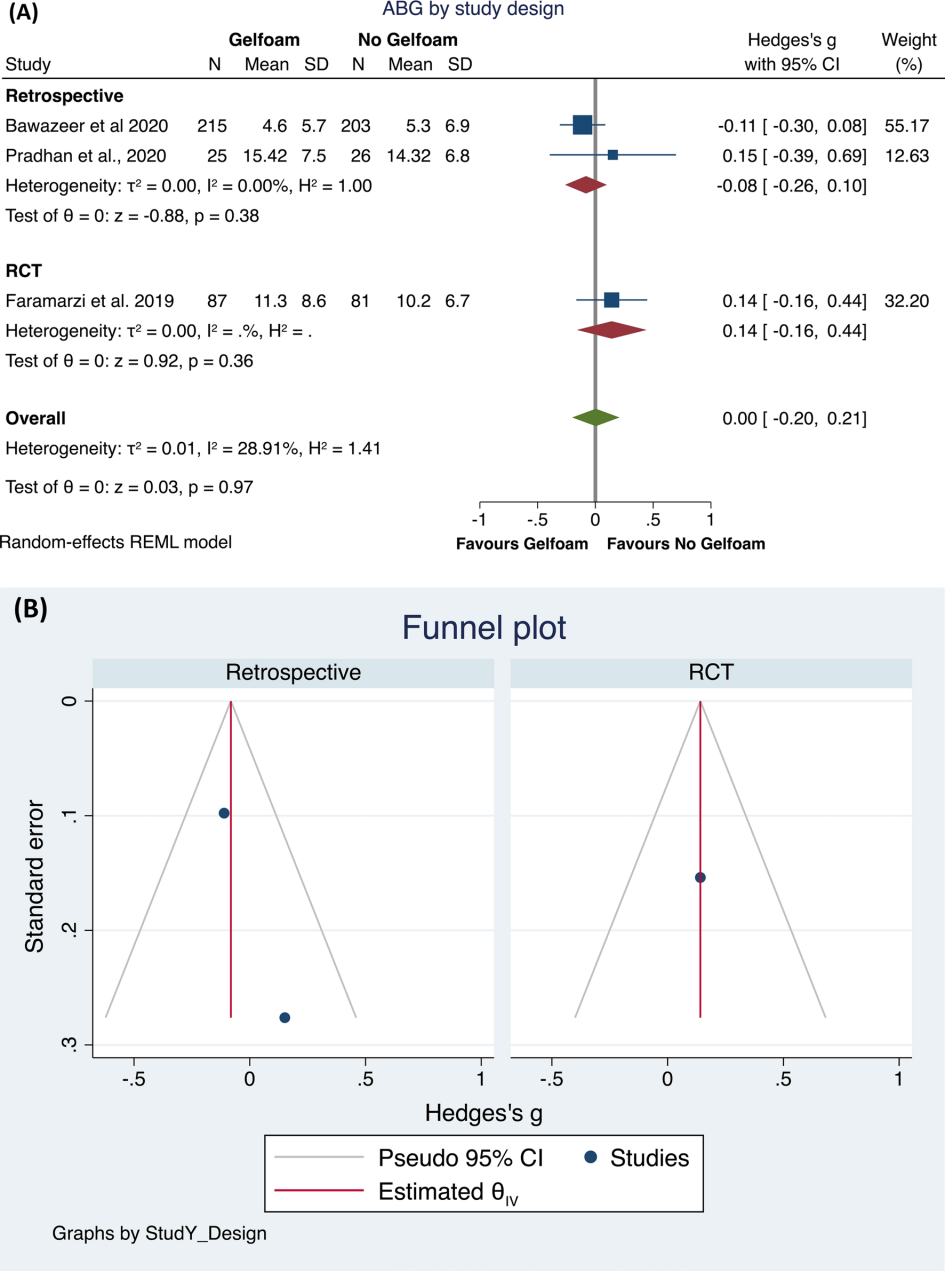
Forest plot (**A**) and Funnel plot (**B**) of the subgroup analysis by Retrospective or RTCs study design in studies comparing the gelfoam and all other treatments in ABG after the intervention

The meta-analysis did not identify differences between the groups (*p* = 0.97), with a low heterogeneity of the results (*I*^2^ = 28.91%; Hedge’s *g* = 0.00, 95% CI − 0.20 to 0.21; Fig. 4A). The Funnel plot and the Egger and Begg’s test did not show small study effects publication bias (Fig. 4B). Then, to check the possible interaction of the study design with these results, we performed a sub-analysis. In the retrospective studies, no statistically significant differences (*p* = 0.38) were found in ABG between the two groups, whereas a very low heterogeneity was found (*I*^2^ = 0.0%). The RTCs included only one study, making analysis impossible (Fig. 5A, B).

### Meta-regression analysis

Since the absence of differences in ABG levels between patients treated with or without fat, we ran a meta-regression analysis to seek potential moderators, such as age and percentage of the female population. Both these parameters did not affect the findings (age: beta = 0.0104; 95% CI − 0.0378 to 0.0586, *p* = 0.673; percentage of female: beta =  − 0.0086; 95%CI − 0.0309 to 0.0136, *p* = 0.447).

Similarly, a meta-regression was performed, to check the possible influence of the age and the percentage of the female population as moderators on the ABG levels in patients treated with or without gelfoam. Again, both these factors did not affect the findings (age: beta = 0.0177; 95% CI − 0.0757 to 0.0404, *p* = 0.550; percentage of female: beta =  − 0.0027; 95% CI − 0.0395 to 0.0340, *p* = 0.884).

## Discussion

Stapes surgery is a successful and widely adopted treatment strategy for otosclerosis [30]. While stapedotomy is the most used technique, many aspects remain to discuss, and individual preferences prevail on universal consensus. Our study aimed to evaluate the influence of different sealing materials on hearing outcomes in stapedotomy. This metanalysis did not find any influence of the fat or the gelfoam sealing materials in conditioning the ABG closure after stapedotomy. These results could be ascribed to the small number of eligible studies.

Only in 3 studies [20, 21, 24] vestibular symptoms were reported: in Angeli’s study [21], after one week, in the HG group two patients referred with dizziness showed up with movement only, and one patient with dizziness regardless of the movements. In contrast, in the control group, five patients presented dizziness triggered by the movements and seven patients independently by movements. Bawazeer et al. [21] reported immediate postoperative vertigo in 25 (11.6%) subjects of the gelfoam group, 17 (8.4%) in the control group, and 3 out of these patients needed hospitalization.

Pradhan et al. [24] evaluated vestibular symptoms with the dizziness handicap inventory (DHI), and, at the end of one week, the fat group showed a mean DHI score significantly (*p* < 0.0001) lower than the gelfoam group (43.92 ± 6.63 vs 51.68 ± 11.38, respectively). However, after 4 weeks no difference was found in the mean DHI score (22.38 ± 9.33 in the fat group, 18.64 ± 6.67 in the gelfoam group). This result was also confirmed at the end of 12 weeks (*p* = 0.30).

Regarding SNHL, Angeli et al. [20] and Faramarzi et al. [22] did not report any case taking into account the average bone-conduction threshold greater than 10 dB both groups. Faramarzi et al. [22] reported nine cases of SNHL at 4 kHz in the fat group and four in the gelfoam one (*p* > 0.05). Pradhan’s [24] and Faramarzi’s [23] studies did not find any case of SNHL in both groups till the last follow-up period.

Bawazeer et al. [21] noted an increase in BC threshold greater than 15 dB in nine patients (4.2%) of the gelfoam and in eight patients (3.9%) of the control group without any statistically significant difference (*p* > 0.05).

Schmerber et al. [25] reported an SNHL with a BC threshold greater than 10 dB in 1.4% and 4.4% in the vein and perichondrium groups, respectively (*p* > 0.05), and an increase in bone conduction level at 4 kHz (> 10 dB) in 8% of cases in the vein group and in 11% of cases in the perichondrium group. Only one case of the perichondrium group presented a dead ear (0.22%).

In our study, we compared the use of fat with all other treatments and the use of the gelfoam with all other treatments. In both cases, the meta-analysis did not identify differences between the groups. The meta-regression analysis confirmed that other moderators, such as age and percentage of the female population, did not influence the results.

Of all the usable materials, fat appears to be a reasonable and practical alternative. Sealing the oval window with small pledgets of fat appears to be a safe and effective alternative, with a clearly favorable cost profile [6]. Similar to the vein, adipose tissue tends to remain stable over the years [10]. Wiet et al. [31] compared fat with other autologous tissues (vein and fascia) in stapedectomy and found that they give comparable and satisfactory results. These conclusions can probably be extended to the stapedotomy as well.

Gelfoam is an easy-to-use material too. Introduced in stapes surgery by House [32] it is probably as effective as other materials, with the advantage of not needing another surgical incision, reducing significantly the time of intervention and the risks connected to harvesting an autologous graft [22]. It is also non-antigenic, easy to handle, and available in almost every context, being a widely used material in otology. However, studies have shown that it can induce adhesions and fibrosis due to fibroblasts' migration in its porous structure, especially in cases of inflamed or denuded mucosa [33]. Based on this alleged risk, Bawazeer et al. [21] retrospectively compared gelfoam use with no sealing and found no significant differences in terms of hearing results and complications. Therefore, the author decided to discontinue the use of gelfoam.

Available sealants can be divided into autologous and heterologous ones. The advantage of autologous materials, such as a vein, perichondrium, and fascia is the cost-effectiveness, compatibility with middle ear mucosa, and similarity with the annular ligament [34], although the harvesting process probably lengthens the surgical time [22]. The utilization of vein graft dates back to stapedectomy described by Shea in the 1950s [35] and it’s still commonly used. A long-term prospective audiometric evaluation study by Vincent et al., [36] demonstrated the stability of the results with vein graft over time. The vein is traditionally harvested from the wrist or dorsum of the hand; alternatively, the superficial temporal vein or its branches can be used, with the added advantages of better cosmetic outcome and working on the same operative site [37]. Like the superficial temporal vein, the tragal or conchal perichondrium has the advantage to be accessible in the same operative field, although there is some concern for the chondrogenic potential of this graft, which can probably be avoided by orienting it properly and not traumatizing it [38].

A comprehensive review by Daou et al. [39] explored the application of Hyaluronic acid (HA) in otology. This review highlighted HA to be generally safe, biocompatible, and degradable in the middle ear, without eliciting a foreign body reaction; it is also stated that its use in stapes surgery decreases postoperative vestibular symptoms and has no significant ototoxicity. HA could act by preventing the blood from entering the inner ear and the perilymph from escaping it. [40]. A certain homogeneity was found regarding the surgical approach used across the studies, the only exception being Pradhan et al. [24] who utilized the endoscopic approach instead of the microscopic one. A recent meta-analysis [41] found the two approaches to be comparable, with less post-operative pain and dysgeusia for endoscopy. At the moment no articles on the influence that the surgical approach could have on the sealing procedure have been produced. While the use of both hands in microscopy could make the positioning of the graft on the fenestra faster, the endoscopic approach allows a wider intraoperative field of view and could render the positioning more precise. These aspects could be the topic of interest for further studies.

Post-operative vertigo is not an uncommon event and can be associated with many causes, such as instability of the footplate or altered labyrinthine fluids equilibrium. Early vertigo episodes, described in 25–30% of the patients [42] are probably related to the surgical procedure and should be distinguished from late episodes. Although no long-term vertigo episodes were reported in the studies we analyzed, it is difficult to conclude with absolute certainty that this is always the case, and more objective evaluation should be used in future studies for the sake of clarity and comparison between studies.

In preventing the occurrence of peri-lymphatic fistula (PLF), the role of oval window sealing is debatable, with some studies showing that a well-calibrated technique is more important [43].

Several authors prefer to not seal the oval window at all [44]. The utility of sealing remains questionable and could represent an inherited residue from past surgical practice, without measurable benefits. Performing this extra step could also add challenge to a procedure that is conceptually simple but difficult to master [45].

Our study showed that hearing outcomes and incidence of vestibular complications are similar regardless of the type of sealant used; the choice of sealing material should be based on the personal preference of the surgeon because no definitive evidence of an advantage in using a sealant over the other is available.

### Strengths and limitations of the study

This systematic review was conducted using transparent methods and a priori defined criteria in accordance with the guidelines of the Cochrane Collaboration and PRISMA. The review protocol was registered in the International Prospective Register of systematic reviews (PROSPERO). To our knowledge, this represents the first metanalysis exploring the effect of the fat or the gelfoam with respect to the other treatments on the ABG closure.

Limitations included a restricted search in language and a few cases due to the unavailability of quality studies. Only two papers were randomized controlled trials while the others were retrospective comparative studies. Another limitation is the relatively short duration of follow-up. The authors of the included studies were not contacted for further information and thus the results are solely based on the published data. Surely the main limitation is the small number of studies introduced that, however, represent the only available in the literature up to date.

## Conclusions

Oval window sealing is often performed during stapedotomy, although no definitive data are available regarding this practice and its convenience over no sealing at all. When sealing is performed, numerous options are offered with acceptable safety and effectiveness profiles; the choice of a sealant should depend on several factors, including surgeon preferences, availability, and cost-effectiveness. Given the abundance of available materials and lack of RCT comparing different choices, no definitive recommendation can be made. Prospective randomized controlled trials using the same drill technique, prosthesis, and approach are warranted in the future to directly compare outcomes and further elucidate the topic.
